# Severity of Vitamin D Deficiency Predicts Mortality in Ischemic Stroke Patients

**DOI:** 10.1155/2019/3652894

**Published:** 2019-05-02

**Authors:** Jarosław Wajda, Maciej Świat, Aleksander J. Owczarek, Aniceta Brzozowska, Magdalena Olszanecka-Glinianowicz, Jerzy Chudek

**Affiliations:** ^1^Dialysis Center in Rybnik, Regional Specialist Hospital No. 3 in Rybnik, Poland; ^2^Department of Neurology with Stroke Unit, Regional Specialist Hospital No. 3 in Rybnik, Poland; ^3^Jan Dlugosz University in Czestochowa, Poland; ^4^Department of Statistics, Department of Instrumental Analysis, Faculty of Pharmacy and Laboratory Medicine in Sosnowiec, Medical University of Silesia, Katowice, Poland; ^5^Health Promotion and Obesity Management Unit, Department of Pathophysiology, Medical Faculty in Katowice, Medical University of Silesia, Katowice, Poland; ^6^Pathophysiology Unit, Department of Pathophysiology, Medical Faculty in Katowice, Medical University of Silesia, Katowice, Poland; ^7^Department of Internal Medicine and Oncological Chemotherapy, Medical Faculty in Katowice, Medical University of Silesia, Katowice, Poland

## Abstract

**Background:**

Vitamin D (VD) deficiency is considered an independent risk factor for death due to cardiovascular events including ischemic stroke (IS). We assessed the hypothesis that decreased levels of 25-hydroxyvitamin D (25-OH-D) are associated with increased risk of mortality in patients with IS.

**Methods:**

Serum 25-OH-D, intact parathyroid hormone (iPTH), and intact fibroblast growth factor 23 (iFGF23) levels were assessed in serum of 240 consecutive patients admitted within the 24 hours after the onset of IS. Mortality data was obtained from the local registry office.

**Results:**

Only three subjects (1.3%) had an optimal 25-OH-D level (30-80 ng/mL), 25 (10.4%) had a mildly reduced (insufficient) level, 61 (25.4%) had moderate deficiency, and 151 (62.9%) had a severe VD deficiency. 20% subjects had secondary hyperparathyroidism. The serum 25-OH-D level was significantly lower than that in 480 matched subjects (9.9 ± 7.1 vs. 21.0 ± 8.7 ng/mL). Of all the patients, 79 (32.9%) died during follow-up observation (44.9 months). The mortality rates (per year) were 4.81 and 1.89 in a group with and without severe VD deficiency, respectively (incidence rate ratio: 2.52; 95% CI: 1.44–4.68). There was no effect of secondary hyperparathyroidism and iFGF23 levels on mortality rates. Age, 25 − OH − D < 10 ng/mL, and functional status (modified Rankin scale) were significant factors increasing the risk of death in multivariable Cox proportional hazard regression test.

**Conclusions:**

Severe VD deficiency is an emerging, strong negative predictor for survival after IS, independent of age and functional status. VD supplementation in IS survivals may be considered due to high prevalence of its deficiency. However, it is uncertain whether it will improve their survival.

## 1. Background

Ischemic stroke occurs in all ages on all continents, often causing irreversible damage to the brain, and is the most common cause of disability in the people over 45 years old. Moreover, strokes are the third leading cause of death [1].

Despite major improvements in primary prevention and acute treatment over the last decades, stroke is still a devastating disease. At the beginning of the 21st century, the age-standardized incidence of stroke in Europe ranged from 95 to 290/100,000 per year, with one-month case-fatality rates ranging from 13 to 35%. Approximately 1.1 million European inhabitants suffered a stroke each year, and IS accounted for approximately 80% of cases [2].

The established risk factors for IS do not explain the occurrence of the disease in one-third of patients [3], justifying further exploration of potential risk factors. Recently, increased frequency of vitamin D (VD) deficiency was found in patients with cardiovascular diseases [4–6], including IS [7], and the association between VD deficiency and increased risk of death due to cardiovascular events was shown [8].

There are a few potential mechanisms that may explain the relationship between VD deficiency and the occurrence of IS. One of the most important is the development of secondary hyperparathyroidism [4–6] with disturbances related to the excess of PTH: insulin resistance, dysfunction of pancreatic beta cells predisposing to type 2 diabetes, hypertension, and dyslipidemia development [5]. Furthermore, activation of the renin-angiotensin system leading to increased blood pressure, stimulation of inflammation, and endothelial dysfunction cause progression of atherosclerosis [6]. In addition, VD may exert a neuroprotective effect by inhibiting inducible nitric oxide synthase (iNOS) that is increased during ischemic events, as well as antioxidant and anti-inflammatory processes [9].

In the present study, we assessed the hypothesis that decreased levels of 25-hydroxyvitamin D (25-OH-D) and calcium-phosphate disturbances are associated with increased risk of mortality in patients with IS.

## 2. Patients and Methods

It was a retrospective cohort study utilizing frozen serum samples that remained after routine laboratory tests in the hospital laboratory, obtained in consecutive patients with acute IS, admitted to the Stroke Unit of the Department of Neurology, Regional Specialist Hospital No. 3 in Rybnik (from January 2013 to August 2015) within 24 hours after the onset of symptoms. The utilization of serum samples and retrieval of data from medical reports without obtaining an informed consent were approved by the Bioethics Committee of the Medical University of Silesia.

Data concerning clinical status, comorbidities, medication, previous ischemic cerebrovascular episodes, cardiovascular risk factors, and laboratory parameters (C-reactive protein, glucose, creatinine, lipid profile, and urinalysis) were obtained from hospital medical files (routine work-up). Follow-up data for subsequent ischemic strokes were obtained from hospital medical records, which were checked quarterly, during and after the observation period for the entire study group, as well as from medical records of general practitioners, and mortality from the Registry Office in Rybnik (last actualization for 31 December 2016).

The diagnosis of acute IS was based on neurological examination and was supported with computed tomography (CT), after exclusion of other causes of acute neurological deficit (including hemorrhagic stroke), and in accordance with current guidelines [10]. Imaging studies were positive for ischemic changes typical for the acute phase of IS and correlated with clinical symptoms. The course of IS was evaluated on the basis of the severity of neurological deficit in the NIHSS (National Institute of Health Stroke Scale) and functional status at discharge using the modified Rankin scale (mRS). Additional studies determined the probable mechanism of IS, according to the TOAST (Trial of Org 10172 in Acute Stroke Treatment) classification [11].

Patients with hemorrhagic stroke, history of cancer, apparent inflammation, and impairment in activities of daily living before the stroke (ADL less than 5 points in the Katz Index) were excluded from the analysis.

### 2.1. Control Group

The group consisted of 480 subjects drawn from a population-based community dwelling cohort of older subjects (PolSenior study [12]) without a history of cancer, matched according to sex, age (73 ± 6 years), occurrence of diabetes (34.8%), and hypertension (81.4%).

### 2.2. Measurements

Additional assessments were performed in stored frozen samples in the laboratory of the Department of Pathophysiology. Serum 25-OH-D (limit of quantification 3 ng/mL) and intact parathyroid hormone (iPTH) levels were assessed by an electrochemiluminescence method on a Cobas E411 analyzer (Roche Diagnostics GmbH, Mannheim, Germany) with inter-assay coefficients of variability below 7.8 and 6.5%, respectively. We measured serum intact fibroblast growth factor 23 (iFGF23) (Immutopics, San Clemente, CA, U.S.) and high-sensitivity (limit of quantification (0.09 mg/L) C-reactive protein (Immundiagnostik AG, Bensheim, Germany) concentrations by ELISA. The mean intra- and interassay coefficients were 4.4% and 6.1% for iFGF23 and <6% and <11.6% for hsCRP, respectively.

Serum phosphorus and calcium were assessed by an automated system (Cobas 111, Roche Diagnostics GmbH, Mannheim, Germany) with interassay coefficients of variability below 2.3 and 1.3%, respectively.

### 2.3. Data Analysis

VD status was categorized by commonly used cutoffs and definitions of serum 25-OH-D: values < 10 ng/mL were categorized as a severe deficiency, between 10 and 19.9 ng/mL as a deficiency, between 20 and 29.9 ng/mL as insufficiency, and those above 30 ng/mL as sufficient 25-OH-D concentrations [13].

Patients with iPTH above 65 pg/mL and normal or low total calcium were diagnosed with secondary hyperparathyroidism.

Estimated glomerular filtration rate (eGFR) was estimated according to the short MDRD formula (Modification of Diet in Renal Disease) [14]. Patients with eGFR < 60 mL/min/1.73 m^2^ or albuminuria in routine urinalysis were considered as having kidney function impairment.

### 2.4. Study End Points

The primary outcome was death, and the secondary outcome was composite end point (CEP) stated as death or a recurrent IS.

### 2.5. Statistical Analysis

Statistical analysis was performed using STATISTICA 10.0 PL (StatSoft, Cracow, Poland), StataSE 13.0 (StataCorp LP, TX, U.S.), and R software. Statistical significance was set at a *p* value below 0.05. All tests were two-tailed and data imputation was not performed. Nominal and ordinal data were expressed as percentages, whilst interval data was expressed as mean value ± standard deviation in the case of a normal distribution or as median with lower and upper quartiles in the case of data with skewed or nonnormal distribution. Distribution of variables was evaluated by the Anderson-Darling test. Homogeneity of variances was assessed by the Levene test.

In order to show survival rate and cumulative hazard estimates according to the follow-up time, Kaplan-Meier curves and Nelson-Aalen estimates were used with a log-rank test to compare survival distribution between two samples. Risk factors of death, as well as CEP, were analyzed with univariable and multivariable stepwise backward Cox proportional hazard regression. All statistically significant factors in univariable analysis were included into a multivariable stepwise backward model (*p* > 0.05 for removal). In the case of age, we used median as a cutoff point.

Schoenfeld residuals were used to test the proportional hazard (PH) assumption. The concordance probability was calculated with Gönen and Heller's *K* concordance coefficient. The functional status was included into the analysis, based on the modified Rankin scale (mRS), at admission equal or higher than 4 pts, while at discharge equal to 5 pts, according to statistical significance of the hazard ratio.

## 3. Results

### 3.1. Study Group Characteristics

Among 240 study patients, 17.5% (*N* = 42) had a previous history of IS, 1.7% (*N* = 4) of transient ischemic attack, and 1.7% (*N* = 4) of intracerebral bleeding. Cardiovascular diseases (particularly hypertension, 84.6%) and diabetes (35.0%) were highly prevalent in the study group (Table 1).

On admission, 27.0% were without or with minor (NIHSS ≤ 4 pts), 54.2% with moderate (5-15 pts), and 18.8% with moderate to severe or severe IS symptoms (≥16 pts). Large vessel occlusion was the most prevalent cause of IS (*N* = 106; 44.2%) followed by lacunar (*N* = 56; 23.3%), embolic stroke (*N* = 55; 22.9%), other classified (*N* = 8; 2.3%), and unclassified (*N* = 15; 6.3%).

### 3.2. VD Levels

Upon hospital admission, only three subjects (1.3%) had a 25-OH-D level within the optimal range, 25 (10.4%) had mildly reduced (insufficient) levels, 61 (25.4%) had moderate deficiency, and 151 (62.9%) had severe deficiency. One patient was supplemented with VD and 2 were treated with bisphosphonates. There was no patient taking calcium-containing supplements.

In the study group, the serum 25-OH-D level was significantly lower than that in the control group (9.9 ± 7.1 vs. 21.0 ± 8.7 ng/mL; *p* < 0.001). In the control group, 163 (34.0%) had mildly reduced (insufficient) levels, 217 (45.2%) moderate deficiency, and only 29 (6.0%) severe deficiency.

There was a greater prevalence of secondary hyperparathyroidism in IS patients (*N* = 48; 20%) than in the control subjects (*N* = 65; 13.7%; *p* < 0.05).

Similar 25-OH-D and iPTH levels were observed in patients regardless of the type of IS (Table 1).

### 3.3. Hospitalization Period

Thrombolysis was performed in 36 patients. During hospitalization, 24 patients (10%) died: 8 (44.4%) with NIHSS > 20, 6 (22.2%) with NIHSS 16–20, 8 (6.1%) with NIHSS 5–15, and 2 (3.2%) with NIHSS 1–4. Similar percentages of fatal outcomes were observed in patients after thrombolysis and treated conservatively (8.3% vs. 10.3%, respectively).

At discharge, 85 patients (39.4% of survivals) had no significant disability (0-1 in mRS), 32 (14.8%) slight disability (2 in mRS), 36 (16.7%) moderate disability (3 in mRS), 31 (14.3%) moderately severe disability (4 in mRS), and 32 (14.8%) sever disability (5 in mRS) (Table 2).

Patients with beneficial outcome (mRS 0-1) had higher 25-OH-D levels than those with fatal outcome (9.0 (*Q*_1_-*Q*_3_: 4.9-14.3) vs. 6.3 (3.4-9.8) ng/mL, *p* < 0.05, respectively). The difference in corresponding iPTH values was not significant (37.1 (*Q*_1_-*Q*_3_: 22.3-52.0) vs. 47.4 (35.2-60.7), *p* = 0.2).

### 3.4. VD and Survival

**Figure 1:**
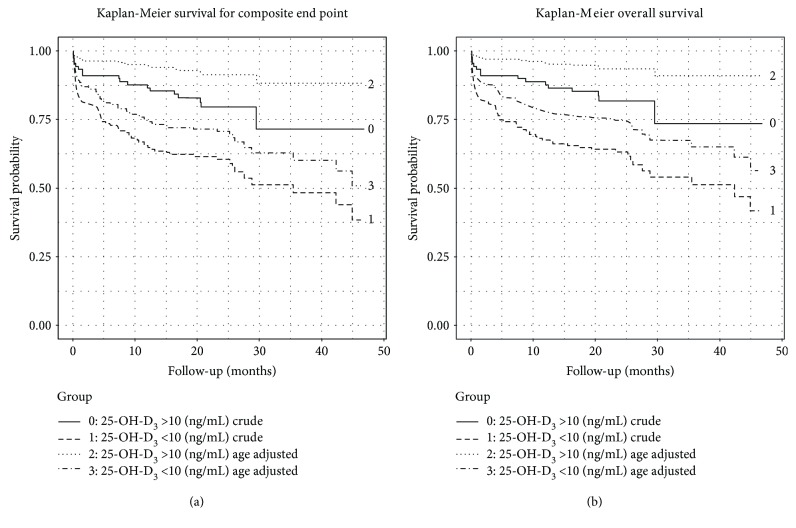
Kaplan-Meier crude and age-adjusted overall survival (a) and probability for composite end point (b) in groups with a 25-OH-D level lower and higher than 10 (ng/mL).

The period of the follow-up was up to 48 months (mean 25 months). The primary outcome, death, was noted in 79 (32.9%) subjects during the period of follow-up. Median survival time and extended mean survival time were 44.9 and 64.5 months, respectively. The mortality rates (per year) were 4.81 and 1.89 in groups with and without severe VD deficiency, respectively, with an incident rate ratio equal to 2.52; (95% CI: 1.44–4.68) (Figure 1). Furthermore, we estimated that 60.4% (95% CI: 30.6%–76.6%) of deaths could be assigned to VD deficiency (population attributable risk (PAR)). Therefore, we estimated it would be necessary to treat 5 (95% CI: 2.9–8.0) subjects with severe VD deficiency to prevent one death (number needed to treat (NNT)).

**Figure 2:**
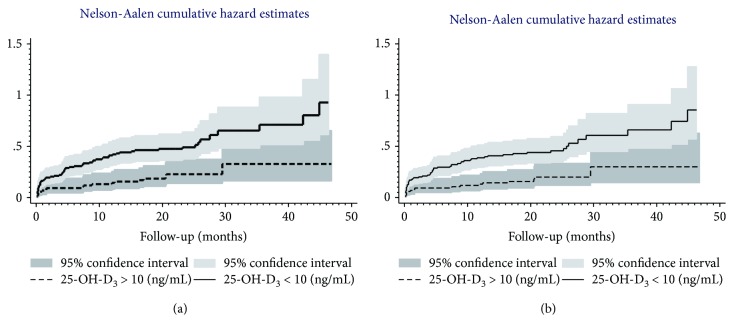
Nelson-Aalen cumulative hazard estimates of death (a) and composite end point (b) in groups with a 25-OH-D level lower and higher than 10 (ng/mL).

The log-rank test for crude as well as for age-adjusted analysis proved differences between survival curves (*χ*^2^ = 10.9, *p* < 0.01 and *χ*^2^ = 9.5, *p* < 0.01, respectively). The difference in survival probability between the groups began after 15.8 months of follow-up. Nelson-Aalen cumulative hazard estimates confirmed a higher hazard rate of death in the group with severe VD deficiency (Figure 2).

According to univariable Cox proportional hazard regression, age over 72 years, female gender, diabetes mellitus and atrial fibrillation occurrence, serum iPTH ≥ 65 pg/mL, 25 − OH − D < 10 ng/mL, and eGFR < 60 mL/min/1.73 m^2^ as well as a patient's mRS ≥ 4 points at admission were risk factors for death. After adjustment for age, female gender, atrial fibrillation occurrence, serum iPTH ≥ 65 pg/mL, and eGFR < 60 mL/min/1.73 m^2^ lost their statistical significance. Patient's mRS at discharge = 5 was the only factor that did not fulfil proportional hazard assumption (also after age adjustment) and was incorporated into the analysis as a time-dependent factor that proved to increase the risk of death (Table 3).

In multivariable stepwise backward Cox proportional hazard regression, age over 72 years, serum 25 − OH − D < 10 ng/mL, and mRS at discharge proved to be statistically significant factors increasing the risk of death (Table 4). Based on Gönen and Heller's *K* concordance coefficient, we may assume that we can correctly identify survival times for pairs of patients 72.2% in crude and 78.3% in an age-adjusted analysis of the time on the basis of the mentioned factors.

### 3.5. VD and Composite End Point (Death or Recurrent Stroke)

Of all patients, 85 (35.4%) had a composite end point (CEP). Lower quartile and extended mean survival time were of 4.6/46.5 and 29.5/137.5 months, respectively. The incidence rates of CEP per year were 4.34 and 1.77 in a group with and without severe VD deficiency, respectively, with an incidence rate ratio equal to 2.42; (95% CI: 1.42–4.34) (Figure 1). Moreover, we estimated that 58.8% (95% CI: 29.7%–76.9%) of CEPs were due to severe VD deficiency (PAR). Therefore, we estimate it would be necessary to treat 5 (95% CI: 2.8–7.9) persons with severe VD deficiency in order to prevent one CEP (NNT).

The log-rank test for crude as well as for age-adjusted analysis proved differences between survival curves (*χ*^2^ = 11.3, *p* < 0.001 and *χ*^2^ = 9.9, *p* < 0.01, respectively). The difference in survival probability for CEP between the groups began after 15.8 months of follow-up. Nelson-Aalen cumulative hazard estimates confirmed a higher hazard rate of CEP in the group with severe VD deficiency.

Table 5 shows results of crude as well as age-adjusted univariable Cox proportional hazard regression analysis. Independent risk factors for CEP were age over 72 years, diabetes, and coronary artery disease occurrence, severe VD deficiency, eGFR > 60 mL/min/1.73 m^2^, and mRS at discharge ≥ 4 points. Furthermore, an increased level of cerebrovascular incidence severity and an increased NIHSS were factors that increased the risk of CEP. With only age adjustment, serum 25 − OH − D < 10 ng/mL, mRS, and cerebrovascular incidence severity proved to be statistically significant. A patient's mRS at discharge = 5 was the only factor that did not fulfil the proportional hazard assumption after age adjustment and was incorporated into the analysis as a time-dependent factor that proved to increase the risk of CEP occurrence.

In multivariable stepwise backward Cox proportional hazard regression, age over 72 years, severe VD deficiency, and mRS proved to be statistically significant factors increasing the risk of CEP occurrence (Table 4.) Based on Gönen and Heller's *K* concordance coefficient, we may assume that we can correctly order survival times for pairs of patients 70.2% in crude and 75.9% in an age-adjusted analysis of the time on the basis of previously mentioned factors.

## 4. Discussion

In this study we show that almost all patients with IS, regardless of age and gender, had VD deficiency or insufficiency and that severe VD deficiency (<10 ng/mL), which is ten times more frequent than in community dwelling older adult matched according to sex, age, diabetes, and hypertension, independently of age and patient condition at discharge from the hospital assessed in mRS, is associated with increased mortality and therefore shorter after IS survival. It should be stressed that lower serum 25-OH-D in patients with unfavourable prognosis were not explained by preexisting cachexia and disability as well as inflammatory or neoplastic diseases, as these patients were excluded from the analysis.

Our results extend the findings of Sun et al. [15], who showed in a prospective observation an elevated (49%) risk of IS in the Nurses' Health Study comparing women in the lowest versus highest tertiles of VD, with the largest (OR = 2.47) for lacunar infarction. The effects of VD deficiency on the occurrence of hypertension and diabetes may explain this strong association because these diseases are considered major risk factors for lacunar stroke [16]. Although recent meta-analysis suggests that both of these clinical conditions are equally associated with all types of IS [17], we demonstrated similar VD, iPTH, and iFGF23 levels regardless of the type of IS. However, the cardiovascular risk factor profile varies according to the different IS subtypes. Atrial fibrillation and ischemic heart disease are more frequent in patients with cardioembolic infarction, hypertension and diabetes in patients with lacunar stroke, and peripheral artery disease, hypertension, diabetes, and chronic obstructive pulmonary disease in patients with atherothrombotic infarction [18].

Notwithstanding, Zhang et al. have recently shown that VD deficiency is an independent prognostic factor of functional outcome in IS patients only without hypertension. The reason why the effect of VD deficiency is restricted to patients without hypertension required further study [19].

Some studies suggest that iPTH and VD are separate risk factors for IS [20]. Certainly, secondary hyperparathyroidism may be related to VD deficiency. However, normocalcemic, VD-sufficient hyperparathyroidism is a frequent and increasing with age finding in the population [21]. We showed that secondary hyperparathyroidism is not a significant predictor of mortality after IS or composite end point (recurrent IS or death). Therefore, the effect of 25-OH-D on high mortality and poor prognosis is probably due to mechanisms beyond calcium-phosphate disturbances. It has been shown that VD affects vasoconstriction by regulating intracellular calcium in vascular smooth muscle cells. Also, arachidonic acid metabolism in the endothelium [21] is disturbed in multiple ways—including modulation of production of proinflammatory cytokines and inhibition of proliferation of inflammatory cells (mostly monocytes), inhibition of macrophage-derived foam cells and angiogenesis, decreasing activation of the renin-angiotensin-aldosterone system, and lowering concentrations of 25-OH-D [21–23]. There were also associations with insulin resistance and low HDL-cholesterol levels [14]. It is unclear which of the above mechanisms is most significant with regard to the effect of VD deficiency on high mortality and poor prognosis.

Recently, serum concentration of fibroblast growth factor 23 (FGF23) has emerged as a novel risk factor for IS. Di Giuseppe et al. [24] reported an increased risk for haemorrhage, but not IS, in the top two FGF23 quartiles. It is further demonstrated by Panwar et al. that FGF23 is not a risk factor for IS [25]. Similarly, our study shows that FGF23 levels do not predict mortality after IS or composite end point (recurrent IS or death).

Although the link between VD deficiency and poor prognosis in IS seems to be undisputed, an unanswered question is whether VD supplementation in deficient subjects may improve the prognosis in IS patients by decreasing the risk of recurrent IS and death from other cardiovascular diseases. Several observational studies have reported a protective effect of VD on short-term functional outcome of IS [7, 26–28]. Lower vitamin D concentration was also proved to be associated with a higher risk of cardiovascular diseases and mortality in the general population [29, 30]; according to our prognostication in this study, effective treatment of 5 persons with severe VD deficiency after stroke may prevent the occurrence of one death or recurrent IS. These estimations are very enthusiastic in nature but are not based on prospective interventional study.

Currently, the only interventional study was performed by Gupta et al. [31]. In this randomized, controlled, open-label trial, the authors showed a 74% reduction in mortality after receiving a single intramuscular injection of 600 000 IU cholecalciferol in the arm followed by oral cholecalciferol 60 000 IU once a month with 1 g of elemental calcium daily.

Before we recommend the use of VD in IS patients to improve IS outcomes and not only calcium and bone metabolism, we first must answer several questions: whether and when to start VD supplementation, how long it should be continued, and what is the optimal level for VD regarding prevention of recurrent IS. This requires large, multicenter, randomized trials covering a wide range of age and race subjects living in various geographical areas.

Our study has some limitations. Despite the lack of sample size calculation, the number of patients, both men and women, was sufficient to analyze the influence of severe VD deficiency on the risk of death but insufficient to assess the effect on the IS recurrence. The reference groups for risk calculation were patients with moderate VD insufficiency and deficiency but included few subjects with sufficient VD levels. Finally, out-of-hospital causes of death were not verified by autopsy. Our findings concerning the prevalence of vitamin D deficiency should not be generalized and restricted to white Caucasians living in the same area.

## 5. Conclusion

Severe VD deficiency is an emerging, strong negative predictor for survival after IS, independent of age and functional status. VD supplementation in stroke survivals may be considered. There is a need to perform a clinical randomized controlled study to demonstrate whether VD supplementation improves an IS patient's survival.

## Figures and Tables

**Table 1:** Patients' characteristics and comparison of groups with three main stroke types.

	All subjects (*N* = 240)	Large vessel stroke (*N* = 106)	Lacunar (*N* = 56)	Embolic (*N* = 55)
Age (years)	72 ± 11	70 ± 12	70 ± 9	78±10^∗∗∗^^,^^^^
Gender (men/women)	109/131	49/57	33/23	17/38
History of previous stroke (*n*/%)	42/17.5	15/14.1	9/16.1	15/27.3
Concomitant diseases				
Hypertension (*n*/%)	203/84.6	88/83.0	48/85.7	50/90.9
Coronary artery disease (*n*/%)	69/28.8	32/30.2	9/16.1	22/40.0^∗∗^
Valvular diseases (*n*/%)	24/10.0	10/9.4	5/8.9	9/16.4
Atrial fibrillation (AF) (*n*/%)	70/29.2	14/13.2	5/8.9	47/85.4^∗∗∗^^,^^^^
Antithrombotic agents in AF patients (*n*/%)	23/32.9	4/28.6	0	19/40.4
Symptomatic atherosclerosis (*n*/%)	101/42.1	52/49.1	19/33.9	23/41.8
Obesity (*n*/%)	44/18.3	19/17.9	11/19.6	12/21.8
Diabetes (*n*/%)	84/35.0	37/34.9	19/33.9	23/41.8
Hypercholesterolemia (*n*/%)	107/44.6	49/46.2	29/51.8	23/41.8
Statin therapy (*n*/%)	92/38.3	41/38.7	18/32.1	25/45.4
Active smokers (*n*/%)	45/18.7	23/21.7	17/30.4	5/9.1^∗∗^
The National Institutes of Health Stroke Scale index (NIHSS-I) at admission	9 ± 6	9 ± 6	6 ± 5	12±7^∗∗∗^
Modified Rankin scale (mRS) at discharge (*n*/%)				
0-1 (no significant disability)	85/26.7	40/37.7	26/46.4	12/21.8
2 (mild disability)	32/13.3	15/14.2	9/16.1	7/12.7
3 (moderate disability)	36/15.0	11/10.4	11/19.6	9/16.4
4-5 (moderately severe and severe disability)	63/26.2	31/29.2	7/12.5	20/36.4
6 (death)	24/10.0	9/8.5	3/5.4	7/12.7
One-year survival (*n*/%)	167/75.0	79/74.5	49/87.5	39/70.9
Two-year survival (*n*/%)	96/70.0	52/68.5	17/87.5	27/63.0
Total cholesterol (mmol/L)	5.1 ± 1.5	5.1 ± 1.4	5.2 ± 1.9	4.8 ± 1.3
LDL-cholesterol (mmol/L)	3.2 ± 1.3	3.3 ± 1.1	3.8 ± 1.4	2.4±0.9^∗∗∗^^,^^^^
HDL-cholesterol (mmol/L)	1.4 ± 0.4	1.4 ± 0.4	1.5 ± 0.3	1.4 ± 0.5
Triglycerides (mmol/L)	1.7 ± 1.0	1.7 ± 0.8	1.8 ± 1.2	1.3 ± 0.8
Creatinine (*μ*mol/L)	89 ± 51	N/A	N/A	N/A
Estimated glomerular filtration rate (eGFR) (mL/min/1. 73 m^2^)	70 ± 25	73 ± 26	72 ± 23	61 ± 22^^^^
eGFR < 60 mL/min/1. 73 m^2^ (*n*/%)	86/35.8	31/29.2	20/35.7	29/52.7^^^^
C-reactive protein (CRP) (mg/L)	4.2 (1.8-9.3)	5.1 (2.0-9.5)	3.3 (1.4-8.4)	3.5 (1.7-8.1)
Calcium (mg/dL)	2.32 ± 0.20	2.31 ± 0.19	2.33 ± 0.20	2.24 ± 0.20
Phosphorus (mg/dL)	1.11 (0.96–1.33)	1.09 (0.95-1.32)	1.22 (0.96-1.48)	1.11 (0.97-1.28)
Intact parathyroid hormone (iPTH) (pg/mL)	40.7 (26.7-56.4)	39.0 (24.4-56.5)	39.5 (21.7-63.0)	42.2 (31.1-61.2)
iPTH > 65 pg/mL (*n*/%)	48/20.0	19/17.9	14/25.0	12/21.8
Intact fibroblast growth factor 23 (iFGF23) (pg/mL)	50.0 (17.6-85.4)	52.1 (21.6-92.2)	66.7 (23.9-84.4)	42.4 (18.5-71.7)
iFGF23 > 50 pg/mL (*n*/%)	120/50.0	54/50.9	34/60.7	25/45.5
25-OH-D (ng/mL)	9.9 ± 7.1	9.7 ± 6.9	11.1 ± 8.2	9.4 ± 6.6
25 − OH − D < 10 ng/mL (*n*/%)	151/62.9	66/62.3	33/58.9	38/69.1

^∗∗^ *p* < 0.01; ^∗∗∗^*p* < 0.001 vs. lacunar stroke. ^^^^*p* < 0.01; ^^^^^*p* < 0.001 vs. large vessel stroke.

**Table 2:** Comparison of subgroups distinguished according to the modified Rankin scale at discharge.

	Modified Rankin scale at discharge			
	0-1 (no significant disability) (*N* = 85)	2-3 (mild/moderate disability) (*N* = 68)	4-5 (moderately severe and severe disability) (*N* = 63)	6 (death) (*N* = 24)
Age (years)	67 ± 11	71 ± 10	76 ± 11^^^^^	79 ± 11^^^^^
Gender (men/women)	48/37	33/35	18/45	10/14
History of previous stroke (*n*/%)	14 (16.5)	14 (20.6)	10 (15.9)	4 (16.7)
Concomitant diseases				
Hypertension (*n*/%)	71 (83.5)	60 (88.2)	54 (85.7)	18 (75.0)
Coronary artery disease (*n*/%)	26 (30.6)	15 (22.1)	20 (31.8)	8 (33.3)
Valvular diseases (*n*/%)	7 (8.2)	7 (10.3)	9 (14.3)	1 (4.2)
Atrial fibrillation (*n*/%)	17 (20.0)	15 (22.1)	27 (42.9)^^^^	11 (45.8)^^^
Obesity (*n*/%)	18 (21.2)	8 (12.8)	16 (25.4)	2 (8.3)
Diabetes (*n*/%)	25 (29.4)	22 (32.3)	26 (41.3)	11 (45.8)
Hypercholesterolemia (*n*/%)	37 (43.5)	35 (51.5)	29 (46.0)	6 (25.0)
Statin therapy (*n*/%)	36 (42.3)	26 (38.2)	22 (34.9)	8 (33.3)
Active smokers (*n*/%)	20 (23.5)	13 (19.1)	11 (17.5)	1 (4.2)
Estimated glomerular filtration rate (eGFR) (mL/min/1. 73 m^2^)	72.9 ± 23.4	74.4 ± 29.2	64.1 ± 21.3	67.1 ± 26.0
eGFR < 60 mL/min/1. 73 m^2^ (*n*/%)	20 (23.5)	22 (32.3)	32 (50.8)^^^^^	12 (50.0)^^^
C-reactive protein (CRP) (mg/L)	3.8 (1.3–9.3)	4.3 (2.6–9.1)	4.2 (1.7–10.6)	4.3 (8.5–8.5)
Calcium (mg/dL)	2.35 ± 0.22	2.31 ± 0.21	2.31 ± 0.18	2.28 ± 0.17
Phosphorus (mg/dL)	1.13 (0.98–1.31)	1.16 (0.94–1.43)	1.11 (0.96–1.32)	1.05 (0.98–1.24)
Intact parathyroid hormone (iPTH) (pg/mL)	36.5 (21.7–49.8)	37.2 (24.9–57.1)	46.9 (31.1–66.9)^^^	47.4 (35.2–60.7)
iPTH > 65 pg/mL (*n*/%)	10 (11.8)	15 (22.1)	17 (27.0)	6 (25.0)
Intact fibroblast growth factor 23 (iFGF23) (pg/mL)	42.3 (10.6–77.2)	58.7 (17.8–92.4)	51.5 (25.3–92.2)	48.2 (19.9–102.1)
iFGF23 > 50 pg/mL (*n*/%)	37 (43.5)	39 (57.4)	33 (52.4)	11 (45.8)
25-OH-D (ng/mL)	9.6 (4.8–14.7)	8.4 (4.9–13.4)	7.2 (3.0–12.1)	6.3 (3.4–9.8)
25 − OH − D < 10 ng/mL (*n*/%)	48 (56.5)	43 (63.2)	42 (66.7)	18 (75.0)^^^
One-year survival *N* (%)	80 (94.1)	58 (85.3)	45 (71.4)^^^^^	—
Two-year survival *N* (%)	60 (91.7)	40 (82.1)	27 (57.8)^^^^^	—

^^^ *p* < 0.05; ^^^^*p* < 0.01; ^^^^^*p* < 0.001 vs. minor stroke (0-1).

**Table 3:** Results of crude and age-adjusted univariable Cox proportional hazard regression for death.

Parameter	Cox proportional hazard—crude analysis					Cox proportional hazard—age adjusted				
	HR	95% CI	*p*	*χ* ^2^	*p*	HR	95% CI	*p*	*χ* ^2^	*p*
Age > 72 years	2.74	1.69–4.43	**<0.001**	1.73	0.19	—	—	—	—	—
Female vs. male	1.67	1.05–2.65	**<0.05**	0.33	0.57	1.27	0.78–2.07	0.34	0.09	0.76
Diabetes	1.79	1.15–2.78	**<0.05**	1.68	0.19	1.57	1.01–2.45	**<0.05**	1.11	0.29
Hypertension	0.87	0.48–1.58	0.65	1.65	0.20	0.71	0.38–1.29	0.26	1.40	0.24
Atrial fibrillation	1.68	1.07–2.64	**<0.05**	0.15	0.70	1.22	0.76–1.96	0.40	0.34	0.55
Ischemic heart disease	1.52	0.96–2.41	0.08	0.02	0.90	1.32	0.83–2.11	0.24	0.01	0.92
Valvular diseases	1.67	0.88–3.17	0.12	0.29	0.59	1.42	0.75–2.71	0.28	0.44	0.51
Previous stroke	1.14	0.67–1.92	0.63	1.59	0.21	0.99	0.59–1.69	0.99	1.57	0.21
Hypercholesterolemia	0.72	0.43–1.19	0.20	0.20	0.65	0.76	0.46–1.26	0.29	0.21	0.64
iPTH ≥ 65 pg/mL	1.64	1.01–2.69	**<0.05**	0.18	0.67	1.41	0.86–2.32	0.17	0.50	0.48
iFGF23 > 50 pg/mL	0.89	0.57–1.39	0.63	0.12	0.73	0.92	0.59–1.44	0.72	0.04	0.84
eGFR < 60 mL/min/1. 73 m^2^	1.71	1.09–2.66	**<0.05**	0.05	0.82	1.26	0.79–2.01	0.33	0.26	0.61
25 − OH − D < 10 ng/mL	2.45	1.41–4.25	**<0.01**	0.40	0.52	2.32	1.33–4.02	**<0.01**	0.26	0.61
CRP > 3.0 mg/L	1.47	0.91–2.38	0.12	0.03	0.86	1.25	0.77–2.03	0.37	0.01	0.97
Modified Rankin scale (mRS) at admission ≥ 4	3.31	1.81–6.03	**<0.001**	2.69	0.10	3.20	1.76–5.85	**<0.001**	2.25	0.13
mRS at discharge = 5	7.64	4.87–12.01	**<0.001**	8.55	**<0.01**	6.47	3.99–10.49	**<0.001**	6.81	**<0.01**
mRS at discharge = 5 with time	1.05	1.01–1.09	**<0.05**	—	**—**	1.04	1.01–1.09	**<0.05**	—	**—**
25-OH-D 20–29.9 ng/mL	2.37	1.15–4.88	**<0.05**	2.41	0.49	2.29	1.11–4.71	**<0.05**	1.79	0.62
25-OH-D 10–19.9 ng/mL	5.38	2.32–12.47	**<0.001**			5.01	2.15–11.67	**<0.001**		
25 − OH − D < 10 ng/mL	11.99	5.23–27.51	**<0.001**			9.11	3.92–21.19	**<0.001**		
NIHSS-I at admission (pts)	1.10	1.06–1.14	**<0.001**	0.33	0.56	1.09	1.04–1.13	**<0.001**	0.19	0.67

**Table 4:** Results of crude and age-adjusted multivariable stepwise backward Cox proportional hazard regression for death and composite end point.

	Crude analysis			Age-adjusted analysis		
	HR	95% CI	*p*	HR	95% CI	*p*
Death						
Age > 72 years	1.80	1.08–2.98	**<0.05**	—	—	—
25 − OH − D < 10 ng/mL	2.14	1.23–3.72	**<0.01**	2.17	1.24–3.77	**<0.01**
Modified Rankin scale (mRS) at admission ≥ 4	2.23	1.22–4.10	**<0.05**	2.23	1.21–4.11	**<0.05**
mRS at discharge = 5	5.79	3.61–9.31	**<0.001**	5.76	3.57–9.29	**<0.001**
Composite end point						
Age > 72 years	1.70	1.09–2.68	**<0.05**	—	—	—
25 − OH − D < 10 ng/mL	2.05	1.21–3.47	**<0.01**	2.07	1.22–3.50	**<0.01**
mRS at admission ≥ 4	1.95	1.05–3.64	**<0.05**	1.94	1.04–3.63	**<0.05**
mRS at discharge = 5	5.08	3.22–8.03	**<0.001**	5.05	3.18–8.01	**<0.001**

**Table 5:** Results of crude and age-adjusted univariable Cox proportional hazard regression for composite end point.

	Cox proportional hazard—crude analysis					Cox proportional hazard—age adjusted				
	HR	95% CI	*p*	*χ* ^2^	*p*	HR	95% CI	*p*	*χ* ^2^	*p*
Age > 72 years	2.40	1.52–3.77	**<0.001**	1.45	0.23	—	—	—	—	—
Female vs. male	1.41	0.91–2.18	0.12	0.38	0.54	1.09	0.69–1.74	0.69	0.14	0.70
Diabetes	1.66	1.08–2.56	**<0.05**	1.98	0.16	1.48	0.96–2.28	0.07	1.42	0.23
Hypertension	0.86	0.48–1.52	0.61	1.23	0.27	0.71	0.40–1.28	0.26	0.99	0.32
Atrial fibrillation	1.47	0.94–2.28	0.09	0.05	0.82	1.10	0.69–1.75	0.67	0.15	0.69
Ischemic heart disease	1.56	1.01–2.44	**<0.05**	0.01	0.91	1.37	0.87–2.16	0.17	0.01	0.92
Valvular diseases	1.72	0.93–3.17	0.08	0.22	0.64	1.49	0.80–2.76	0.21	0.34	0.56
Previous stroke	1.02	0.60–1.71	0.95	1.28	0.26	0.91	0.54–1.53	0.72	1.27	0.26
Hypercholesterolemia	0.81	0.49–1.33	0.40	0.09	0.76	0.85	0.52–1.39	0.52	0.11	0.74
iPTH ≥ 65 pg/mL	1.56	0.97–2.52	0.07	0.27	0.60	1.37	0.84–2.22	0.20	0.63	0.43
iFGF23 > 50 pg/mL	0.86	0.56–1.31	0.48	0.02	0.89	0.88	0.57–1.35	0.57	0.01	0.97
eGFR < 60 mL/min/1. 73 m^2^	1.63	1.06–2.51	**<0.05**	0.05	0.83	1.25	0.79–1.96	0.33	0.25	0.61
25 − OH − D < 10 ng/mL	2.38	1.41–4.01	**<0.01**	0.53	0.47	2.26	1.34–3.81	**<0.01**	0.37	0.54
CRP > 3.0 mg/L	1.47	0.92–2.35	0.10	0.04	0.84	1.27	0.80–2.04	0.31	0.01	0.97
Modified Rankin scale (mRS) at admission ≥ 4	3.04	1.67–5.52	**<0.001**	3.14	0.07	2.96	1.63–5.39	**<0.001**	2.64	0.10
mRS at discharge = 5	6.54	4.24–10.09	**<0.001**	9.26	**<0.01**	5.68	3.57–9.03	**<0.001**	7.91	**<0.01**
mRS at discharge = 5 with time	1.04	1.01–1.08	**<0.05**	—	—	1.04	0.99–1.08	0.08	—	—
25-OH-D 20–29.9 ng/mL	1.88	0.99–3.56	0.05	2.88	0.41	1.82	0.96–3.46	0.07	2.24	0.52
25-OH-D 10–19.9 ng/mL	3.96	1.83–8.58	**<0.001**			3.72	1.71–8.10	**<0.01**		
25 − OH − D < 10 ng/mL	8.87	4.14–19.00	**<0.001**			6.96	3.20–15.11	**<0.001**		
NIHSS-I at admission (pts)	1.08	1.04–1.13	**<0.001**	0.43	0.51	1.07	1.03–1.12	**<0.01**	0.30	0.58

## Data Availability

The datasets analysed during the current study are not publicly available due to the continuation of analyses but are available from the corresponding author on reasonable request.

## Abbreviations

IS: Ischemic stroke
VD: Vitamin D
25-OH-D: 25-Hydroxyvitamin D
iPTH: Intact parathyroid hormone
iFGF23: Intact fibroblast growth factor 23
iNOS: Inducible nitric oxide synthase
CT: Computed tomography
NIHSS: National Institute of Health Stroke Scale
mRS: Modified Rankin scale
TOAST: Trial of Org 10172 in Acute Stroke Treatment
ADL: Activities of daily living
eGFR: Estimated glomerular filtration rate
MDRD: Modification of diet in renal disease
pH: Proportional hazard
PAR: Population attributable risk
NNT: Number needed to treat
CEP: Composite end point
HDL-cholesterol: High-density lipoprotein cholesterol.
